# Impact of Overweight/Obesity on Clinical Outcomes of Patient with Vasospastic Angina: From the Vasospastic Angina in Korea Registry

**DOI:** 10.1038/s41598-020-61947-7

**Published:** 2020-03-18

**Authors:** Min-Ho Lee, Sang-Ho Jo, Seongsoon Kwon, Byung Won Park, Duk Won Bang, Min Su Hyon, Sang Hong Baek, Seung Hwan Han, Sung-Ho Her, Dong Il Shin, Sung-Eun Kim, Won-Woo Seo

**Affiliations:** 1 Division of Cardiology, Department of Internal Medicine, Soonchunhyang University Seoul Hospital, Seoul, South Korea; 2 Division of Cardiology, Department of Internal Medicine, Hallym University Pyeongchon Sacred Heart Hospital, Anyang, South Korea; 3 Division of Cardiology, Department of Internal Medicine, Seoul St. Mary’s Hospital, The Catholic University of Korea, Seoul, South Korea; 4 Division of Cardiology, Department of Internal Medicine, Gil Medical Center, Gachon University, Incheon, South Korea; 5 Division of Cardiology, Department of Internal Medicine, Daejeon St. Mary’s Hospital, The Catholic University of Korea, Daejeon, South Korea; 6 Division of Cardiology, Department of Internal Medicine, Pyeongtaek St. Mary’s Hospital, Pyeongtaek, South Korea; 7 Division of Cardiology, Department of Internal Medicine, Hallym University Kangdong Sacred Heart Hospital, Seoul, South Korea

**Keywords:** Ischaemia, Interventional cardiology

## Abstract

Obesity is associated with a high risk of morbidity and mortality in the general population and is a major independent risk factor for cardiovascular disease. We sought to evaluate the effect of overweight/obesity on clinical outcomes of patients with vasospastic angina (VA) at 1-year follow-up. The VA-KOREA (Vasospastic Angina in Korea) registry was a cohort of 11 centers from 2010 to 2015. The primary endpoint was a composite of cardiac death (CD), new-onset arrhythmia, and acute coronary syndrome (ACS). Using the body mass index (BMI) cut-off for Asians, 517 patients with definite VA were divided into either an overweight/obese (BMI ≥ 23 kg/m^2^) group (n = 378) or a normal weight (BMI 18.5–22.9 kg/m^2^) group (n = 139). The overweight/obese group showed a significantly lower rate of the primary endpoint composite (2.4% vs 7.9%, *p* = 0.004) and ACS (0.8% vs 4.3%, *p* = 0.007) than the normal weight group in the crude population. Similarly, in propensity-score matched analysis, the overweight/obese group showed a significantly lower rate of the primary endpoint composite (2.3% vs 8.4%, *p* = 0.006) and ACS (1.1% vs 4.6%, *p* = 0.035) than the normal weight group. However, there were no significant differences in CD and new-onset arrhythmia between the two groups in both the crude and propensity-score matched population. Independent predictors of the primary endpoint were overweight/obesity and dyslipidemia. In patients with VA, the overweight/obese group was associated with a favorable 1-year primary endpoint and the difference was mainly driven by the lower rate of ACS compared with the normal weight group.

## Introduction

Obesity has increased in epidemic proportions over recent decades and represents a growing public health issue. Obesity is associated with a high risk of morbidity and mortality in the general population and is a major independent risk factor for various manifestations of cardiovascular disease (CVD), including hypertension, coronary artery disease (CAD), and heart failure^1–4^. In the Framingham Heart Study cohort, being overweight was associated with a 3-year decrease in life expectancy and obesity with a 6 to 7-year decrease in life expectancy, compared with normal weight^5^. Obesity was also linked to an 81% increased risk for premature death for men and an 115% increased risk for premature death for women^5^. Previous studies have also reported obesity to be an independent predictor of adverse cardiac events after percutaneous coronary intervention in patients with CVD^6,7^.

Despite the numerous adverse effects of obesity on general and CV health, multiple studies have demonstrated that obese patients generally have a more favorable prognosis than do their leaner counterparts^3,4,8,9^. This inverse relation between obesity and CV outcomes is known as the “obesity paradox”.

Body mass index (BMI) is a measure of weight adjusted for height and is often considered as a surrogate parameter for the assessment of obesity. Since BMI is the most readily measured parameter of obesity in clinical practice, the “obesity paradox” has been most commonly demonstrated when using BMI to define obesity^8^. However, unlike the relationship between overweight/obesity and other forms of CAD, the impact of overweight/obesity on clinical outcomes in patients with vasospastic angina (VA) has not been evaluated to date. Therefore, we sought to carefully evaluate the relationship between overweight/obesity and clinical outcomes in patients with VA at 1-year follow-up in a cohort of patients from the VA-KOREA (Vasospastic Angina in Korea) registry.

## Results

### Baseline characteristics

The demographic and angiographic characteristics at baseline are presented in Table 1. The overweight/obese group had more unfavorable demographic characteristics such as a significantly higher frequency of dyslipidemia (17.5% vs 10.1%, *p* = 0.040) and a higher LDL cholesterol level (106.50 mg/dL vs 92.00 mg/dL, *p* = 0.048) compared with the normal weight group. Other baseline characteristics were not different between the two groups.

**Table 1:** Baseline characteristics of the crude population.

	Overweight/obese group (n = 378)	Normal weight group (n = 139)	p value
Age (years)	56.17 ± 10.75	57.12 ± 10.73	0.378
Male, n (%)	274 (72.5%)	106 (76.3%)	0.432
Risk factors of CAD			
Hypertension, n (%)	179 (47.4%)	53 (38.1%)	0.073
Diabetes mellitus, n (%)	44 (11.7%)	8 (5.8%)	0.068
Dyslipidemia, n (%)	66 (17.5%)	14 (10.1%)	0.040
History of CAD, n (%)	56 (14.9%)	23 (16.7%)	0.679
Current smoker, n (%)	130 (34.5%)	55 (39.9%)	0.300
History of thyroid disease, n (%)	9 (2.4%)	2 (1.4%)	0.735
Biochemical parameters			
Creatinine (mg/dL)	0.82 (0.68–0.97)	0.79 (0.66–0.90)	0.248
Troponin I (ng/dL)	0.02 (0.01–0.09)	0.01 (0.01–0.04)	0.762
CK-MB (ng/dL)	1.12 (0.71–2.19)	1.61 (0.90–5.20)	0.946
NT-proBNP (pg/mL)	38.85 (20.73–71.78)	50.50 (23.90–122.50)	0.202
hsCRP (mg/L)	0.09 (0.04–0.28)	0.08 (0.03–0.25)	0.145
Total cholesterol (mg/dL)	174.43 ± 35.48	171.90 ± 37.71	0.501
LDL cholesterol (mg/dL)	106.50 (82.03–121.50)	92.00 (74.00–111.10)	0.048
LVEF (%)	63.85 (59.65–67.85)	63.00 (58.70–67.40)	0.810
Medications prior to enrollment			
CCBs, n (%)	96 (25.5%)	28 (20.7%)	0.294
Beta-blockers, n (%)	35 (9.3%)	10 (7.5%)	0.597
RAS inhibitors, n (%)	88 (23.3%)	26 (19.5%)	0.399
Statins, n (%)	64 (17.0%)	19 (14.4)	0.584

Abbreviations: CAD = coronary artery disease; CK–MB = creatine kinase-MB; NT-proBNP = N-terminal pro-B-type natriuretic peptide; hsCRP = high-sensitivity C-reactive protein; LDL = low-density lipoprotein; LVEF = left ventricular ejection fraction; CCB = calcium-channel blocker; RAS = renin-angiotensin system.

### Clinical outcomes in the crude population

**Figure 1:**
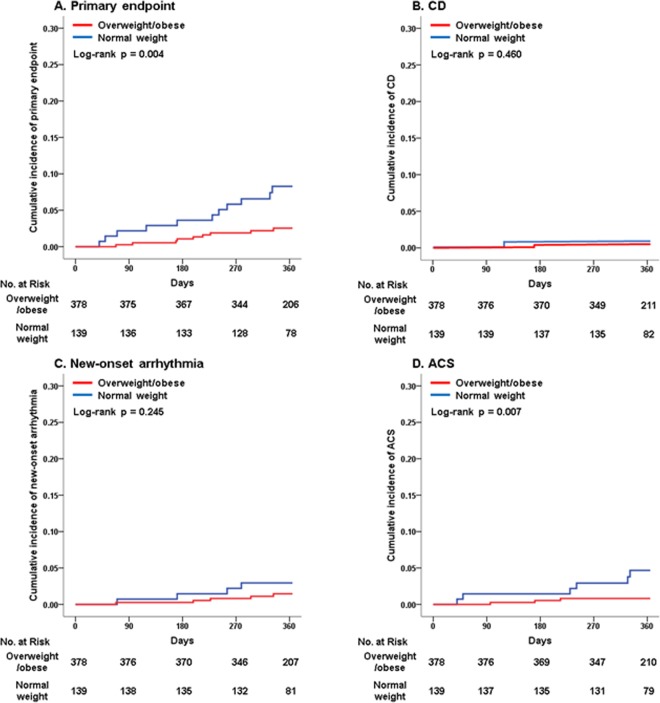
Kaplan-Meier curves of clinical outcomes at 1-year follow-up in the crude population. (**A**) Primary endpoint; (**B**) CD; (**C**) New-onset arrhythmia; (**D**) ACS. Abbreviations: CD = cardiac death; ACS = acute coronary syndrome.

The primary and secondary outcomes in the crude population at 1-year follow-up are shown in Table 2 and Fig. 1. The rate of the primary endpoint was significantly lower in the overweight/obese group than in the normal weight group (2.4% vs 7.9%, *p* = 0.004), which was mainly attributed to the lower rate of acute coronary syndrome (ACS) in the overweight/obese group (0.8% vs 4.3%, *p* = 0.007). However, there were no differences in rates of cardiac death (CD) (0.3% vs 0.7%, *p* = 0.460) and new-onset arrhythmia (1.3% vs 2.9%, *p* = 0.245) between the overweight/obese and normal weight groups.

**Table 2:** Clinical outcomes at 1-year follow-up.

Crude population			
	Overweight/obese group (n = 378)	Normal weight group (n = 139)	p value
Primary endpoint	9 (2.4%)	11 (7.9%)	0.004
CD	1 (0.3%)	1 (0.7%)	0.460
New-onset arrhythmia	5 (1.3%)	4 (2.9%)	0.245
ACS	3 (0.8%)	6 (4.3%)	0.007
**Propensity-score matched population**			
	**Overweight/obese group (n = 262)**	**Normal weight group (n = 131)**	**p value**
Primary endpoint	6 (2.3%)	11 (8.4%)	0.006
CD	0 (0.0%)	1 (0.8%)	0.157
New-onset arrhythmia	3 (1.1%)	4 (3.1%)	0.192
ACS	3 (1.1%)	6 (4.6%)	0.035

Abbreviations: CD = cardiac death; ACS = acute coronary syndrome.

### Clinical outcomes in the propensity-score matched population

We made propensity-score matching to minimize allocation bias and better represent the treatment impact of overweight/obesity in VA. After 2:1 propensity-score matching, 262 of 378 patients in the overweight/obese group (69.3%) were successfully matched to 131 patients in the normal weight group. The two groups were well-balanced in their baseline characteristics after matching (Table 3).

**Table 3:** Baseline characteristics of the propensity-score matched population.

	Overweight/obese group (n = 262)	Normal weight group (n = 131)	p value
Age (years)	56.19 ± 10.80	57.31 ± 10.55	0.326
Male, n (%)	201 (76.7%)	(76.3%)	>0.999
Risk factors of CAD			
Hypertension, n (%)	102 (38.9%)	50 (38.2%)	0.913
Diabetes mellitus, n (%)	14 (5.3%)	6 (4.6%)	0.813
Dyslipidemia, n (%)	29 (11.1%)	14 (10.7%)	>0.999
History of CAD, n (%)	44 (16.8%)	19 (14.5%)	0.662
Current smoker, n (%)	104 (39.7%)	54 (41.2%)	0.827
History of thyroid disease, n (%)	5 (1.9%)	2 (1.5%)	0.787
Biochemical parameters			
Creatinine (mg/dL)	0.83 (0.67–0.96)	0.79 (0.66–0.91)	0.184
Troponin I (ng/dL)	0.02 (0.01–0.10)	0.01 (0.01–0.04)	0.644
CK-MB (ng/dL)	1.04 (0.58–2.04)	1.21 (0.77–5.00)	0.687
NT-proBNP (pg/mL)	49.27 (21.94–120.32)	50.50 (24.15–113.65)	0.262
hsCRP (mg/L)	0.09 (0.04–0.29)	0.05 (0.03–0.25)	0.308
Total cholesterol (mg/dL)	174.12 ± 34.38	173.03 ± 37.63	0.786
LDL cholesterol (mg/dL)	108.50 (84.50–122.25)	88.00 (73.00–111.05)	0.101
LVEF (%)	64.10 ± 6.34	63.63 ± 6.64	0.472
Medications prior to enrollment			
CCBs, n (%)	58 (22.1%)	25 (19.1%)	0.515
Beta-blockers, n (%)	21 (8.0%)	9 (6.9%)	0.841
RAS inhibitors, n (%)	59 (22.5%)	26 (19.8%)	0.604
Statins, n (%)	43 (16.4%)	19 (14.5%)	0.662

Abbreviations: CAD = coronary artery disease; CK-MB = creatine kinase-MB; NT-proBNP = N-terminal pro-B-type natriuretic peptide; hsCRP = high-sensitivity C-reactive protein; LDL = low-density lipoprotein; LVEF = left ventricular ejection fraction; CCB = calcium-channel blocker; RAS = renin-angiotensin system.

**Figure 2:**
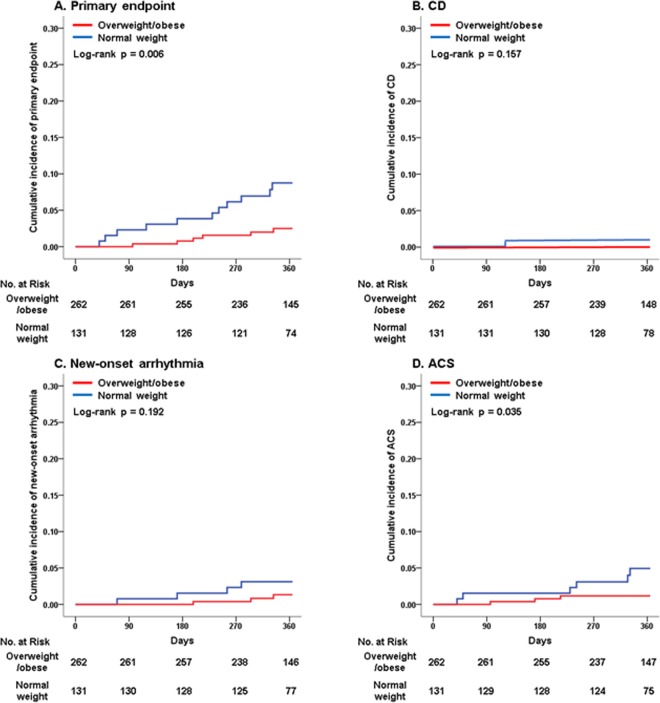
Kaplan-Meier curves of clinical outcomes at 1-year follow-up in the propensity-score matched population. (**A**) Primary endpoint; (**B**) CD; (**C**) New-onset arrhythmia; (**D**) ACS. Abbreviations: CD = cardiac death; ACS = acute coronary syndrome.

The primary and secondary outcomes of the propensity-score matched population were similar to those of the crude population (Table 2 and Fig. 2). Compared with the normal weight group, the overweight/obese group had a significantly lower rate of the primary endpoint (2.3% vs 8.4%, *p* = 0.006) and ACS (1.1% vs 4.6%, *p* = 0.035). However, there were no differences in CD (0.0% vs 0.8%, *p* = 0.157) and new-onset arrhythmia (1.1% vs 3.1%, *p* = 0.192) between the overweight/obese and normal weight group.

### Independent predictors of primary endpoint

We conducted Cox-proportional hazard regression analysis to identify independent predictors of the primary endpoint for VA (Table 4). The following 12 variables were included in the model: overweight/obesity, old age (≥65 years), sex, hypertension, diabetes mellitus, dyslipidemia, history of CAD, current smoking, history of thyroid disease, calcium-channel blocker (CCB)s, and beta-blockers and renin-angiotensin system (RAS) inhibitors. Significant independent predictors were: (1) overweight/obesity (HR 0.258, 95% CI 0.106–0.629, p = 0.003), and (2) dyslipidemia (HR 3.732, 95% CI 1.471–9.469, *p* = 0.006).

**Table 4:** Independent predictors of primary endpoint at 1-year follow-up.

	Univariate analysis		Multivariate analysis	
	HR (95% CI)	p value	HR (95% CI)	p value
Overweight/obesity	0.300 (0.124–0.724)	0.007	0.258 (0.106–0.629)	0.003
Old age (≥65 years)	0.573 (0.168–1.957)	0.375		
Male	1.446 (0.483–4.326)	0.509		
Hypertension	0.816 (0.334–1.996)	0.656		
Diabetes mellitus	0.477 (0.064–3.562)	0.470		
Dyslipidemia	3.046 (1.215–7.635)	0.018	3.732 (1.471–9.469)	0.006
History of CAD	1.377 (0.460–4.119)	0.567		
Current smoking	1.845 (0.768–4.434)	0.171		
History of thyroid disease	2.499 (0.335–18.674)	0.372		
CCBs	1.671 (0.667–4.189)	0.273		
Beta-blockers	1.169 (0.271–5.038)	0.834		
RAS inhibitors	0.864 (0.289–2.585)	0.794		

Abbreviations: CAD = coronary artery disease; CCB = calcium-channel blocker; RAS = renin-angiotensin system.

### Subgroup analysis

**Figure 3:**
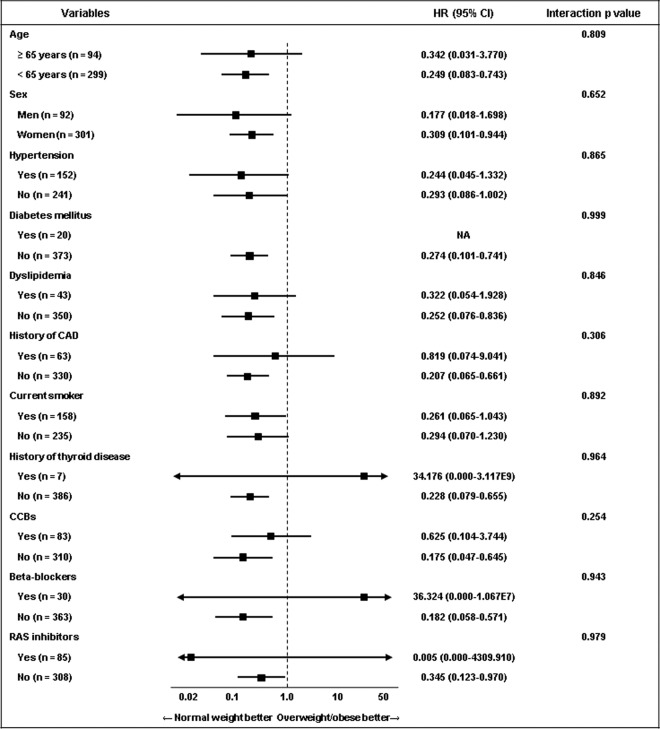
Subgroup analysis for Primary endpoint in the propensity-score matched population. Abbreviations: CAD = coronary artery disease; CCB = calcium-channel blocker; RAS = renin-angiotensin system.

Subgroup analysis regarding the primary endpoint was performed according to old age (≥65 years), sex, hypertension, diabetes mellitus, dyslipidemia, history of CAD, current smoker, history of thyroid disease, CCBs, beta-blocker, and RAS inhibitors. There were no significant differences in the primary endpoint between the overweight/obese and normal weight groups, and these results were consistent across all subgroups, without any significant interaction p value (Fig. 3).

## Discussion

To the best of our knowledge, this is the first study to assess the effect of overweight/obesity in patients with VA using the VA-KOREA registry, a large observational cohort, to describe the clinical outcomes of VA. The major findings of the present analyses are: (1) the overweight/obese group had a lower rate of the primary endpoint and ACS than the normal weight group at 1-year follow-up, even after adjustment (Cox-proportional) or propensity-score matching; (2) however, there were no significant differences in CD and new-onset arrhythmia between the two groups in both the crude and propensity-score matched population; and (3) overweight/obesity and dyslipidemia were independent predictors of the primary endpoint in patients with VA. These findings are largely consistent with past reports of phenomenon of the “obesity paradox” in the CAD setting^3,4,8,9^.

Despite the negative influence of obesity on many risk factors associated with CAD, such as hypertension, diabetes mellitus, and dyslipidemia^10^, once CAD is established, overweight and obese patients actually have a better prognosis. Previously, regarding stable CAD, Hastie *et al*. showed that overweight patients had a significantly lower risk of all-cause mortality than did normal weight patients over a 5-year follow-up period (HR 0.59, 95% CI 0.39–0.90, 95%, *p* = 0.014) in 4,880 patients undergoing elective percutaneous coronary intervention^11^. Furthermore, in the APPROACH registry, which included 31,021 patients with established CAD with a median follow-up time of 46 months, obese patients had reduced mortality compared with normal BMI patients, whether treated with medical management only, percutaneous coronary intervention, or coronary artery bypass grafting^12^.

The existence of an “obesity paradox” has also been demonstrated in ACS as well as in stable CAD. In the PREMIER and TRIUMPH registries of 6,359 patients with acute myocardial infarction (MI), BMI was negatively associated with mortality rate at 1-year (normal, 9.2%; overweight, 6.1%; obese, 4.7%; morbidly obese; 4.6%; *p* < 0.001), which persisted after multivariable adjustment^13^. Neeland *et al*. also demonstrated that patients with normal weight were at higher mortality risk (HR 1.30, 95% CI 1.15–1.47) compared with patients with Class I obesity (BMI 30–34 kg/m^2^) in 19,499 elderly patients with ST-segment elevation MI from the NCDR-ACTION registry^14^.

Although historically VA is considered a relatively benign category of CAD, it is nowadays known to have a significant role in the pathogenesis of various manifestations of CAD^15,16^. Our data corroborate these prior reports of an “obesity paradox” in the setting of other forms of CAD and extend the observations to a large VA cohort. It is noteworthy that overweight/obesity was associated with a more favorable prognosis in those patients with VA. Since the pathophysiology of VA is not fully elucidated and the mechanisms of the “obesity paradox” in VA are not immediately obvious, we can only speculate on the possible mechanisms, as follows.

As obesity advances, a low-grade systemic pro-inflammatory state is induced by the hypertrophied adipocytes and this state is fortified by macrophage recruitment to adipose tissue^2^. Also, obesity leads phenotypic changes in macrophages, which gather in adipose tissue and express genes of the M1 type, which produce inducible nitric oxide synthase (NOS)^2^. Because endothelial dysfunction, caused by abnormalities of endothelial NOS in coronary artery segments, is considered to be the main pathophysiological cause of the coronary artery spasm, these changes might be part of a protective mechanism against VA. Adipose tissue also produces soluble tissue necrosis factor receptor which is considered to neutralize the harmful effects of tumor necrosis factor-alpha on the myocardium^17^. These favorable effects may compensate the deleterious effects of obesity^11^. Additionally, the increased nutritional and metabolic reserves associated with obesity, and its enhanced protection against endotoxin/inflammatory cytokines, might play a role in modulating disease progression and confer a favorable prognosis in obese patients^18^. On the other hand, the “obesity paradox” may simply be due to confounding factors or biases^3,12,19^. For example, overweight/obese patients are younger and tend to present with co-morbidities earlier than those patients with a normal BMI, and this may be a confounding factor^12^. Our findings that overweight/obese patients were younger and showed higher rates of hypertension, diabetes mellitus and dyslipidemia, support this notion. In addition, there appears to be a lead time bias whereby overweight/obese patients are investigated and treated at an earlier stage in the disease process^19^.

This study has several limitations. First, there may be an allocation bias based on an unequal distribution of baseline characteristics, since this was a non-randomized, observational study using a paucity of data. Second, BMI was measured only once, at the time of admission, and weight change after discharge and during follow-up periods was not routinely measured. Third, although BMI is widely used as an epidemiological measure of obesity, it is not ideal and does not give detailed information on body composition or fat distribution. Fourth, measures of fitness and physical activity level were not recorded in this registry and might be unmeasured confounders. Finally, as this study only enrolled Koreans and BMI was categorized according to the Asian-Pacific cutoff points, these results might not apply in other racial/ethnic populations.

In summary, our findings from the VA-KOREA registry show that overweight/obesity is associated with a decreased risk for the primary endpoint and ACS in patients with VA, and the beneficial effect of overweight/obesity persists even after adjustment for baseline characteristics and propensity-score matching. Thus, we help to clarify the concept of the “obesity paradox” in a large VA cohort.

## Methods

### Study population

**Figure 4:**
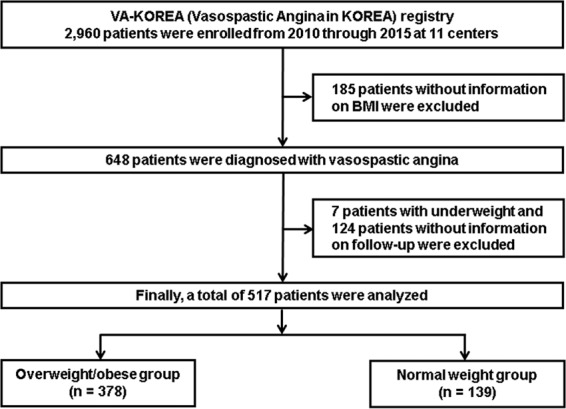
Study flow chart. Abbreviations: VA-KOREA = Vasospastic Angina in KOREA; BMI = body mass index.

The study flow chart is presented in Fig. 4. The VA-KOREA registry is a nation-wide prospective, observational registry of demographic, angiographic, and prognostic data from patients who underwent the ergonovine provocation tests in 11 cardiovascular centers in South Korea^20,21^. Patients were included who were determined by clinicians to have suspicious symptoms and thus underwent coronary angiography (CAG) with the ergonovine provocation test^20,21^. The VA-KOREA registry included 2,960 consecutive patients from May 2010 to June 2015. Of these, 185 patients were excluded due to the unavailability of their BMI. A total of 648 patients were diagnosed with VA; of these, 131 patients were excluded because they were underweight (n = 7) or lost to follow-up (n = 124). Thus, the data from 517 patients with VA were used for the final analysis. Of these, 378 patients were classified into an overweight/obese group and 139 patients into a normal weight group. This study was approved by Hallym University Sacred Heart Hospital Institutional Review Board (Approved No. 2010-I007) and was conducted according to the principals of the Declaration of Helsinki. All patients gave their written informed consent.

### CAG and provocation test for VA

We diagnosed VA on the basis of the criteria set out in the Guidelines for Diagnosis and Treatment of Patients with Vasospastic Angina published by the Japanese Circulation Society^22^. For the ergonovine provocation test, we used intracoronary infusion of ergonovine after baseline CAG. Incremental doses of 20 μg (E1), 40 μg (E2), and 60 μg (E3) were injected into the left coronary artery (LCA). If coronary spasm in the LCA was not provoked, incremental doses of 10 μg (E1), 20 μg (E2), and 40 μg (E3) were then injected into the right coronary artery^20,23^. Once spasm was provoked, intracoronary nitrate was injected. The vasoactive drugs were discontinued at least 48 h before CAG.

VA was diagnosed if patients had a significant vasospasm, defined as total or subtotal occlusion (>90% luminal diameter narrowing) of the coronary arteries after intracoronary ergonovine injection, in addition to the presence of ischemic symptoms and/or electrocardiographic changes^22^. An ischemic ECG change was defined as an ST-segment elevation or depression >0.1 mV or a negative U-wave in at least two related leads^22,24^. Spontaneous spasm, which was defined as a >90% diameter stenosis on baseline CAG relieved after intracoronary nitrate injection, was also included in the diagnosis of VA^20,24^.

### Study endpoints and definition

The primary endpoint was a composite of CD, new-onset arrhythmia, and ACS. The secondary endpoints included individual components of the primary endpoint. CD was defined as any death due to a proximate cardiac cause such as MI, low-output failure, fatal arrhythmia, and death from unknown causes^24^. Patients who presented for the first time during the follow-up with arrhythmias such as atrial or ventricular tachycardia/fibrillation, symptomatic premature beats, sick-sinus rhythm, and atrioventricular block, were considered to have new-onset arrhythmia^20^. ACS was defined as recurrent or continuous chest pain lasting more than 20 min with ischemic ECG changes or elevation of MI cardiac markers^21^. MI was defined as the presence of newly-developed Q wave, raised myocardial muscle creatine kinase, troponin I or T above the normal range, and typical ischemic symptoms with accompanying ST elevation^25^. Hypertension was defined as either blood pressure ≥140/90 mmHg or the current use of anti-hypertensive medications. Dyslipidemia was defined as either total cholesterol ≥240 mg/dL or the current use of dyslipidemia medications.

### BMI and categorization

According to the National Heart Lung and Blood Institute^26^, BMI is calculated as weight in kilograms divided by the square of the height in meters (kg/m^2^) and is categorized into four groups according to the Asian-Pacific cutoff points: underweight (<18.5 kg/m^2^), normal weight (18.5–22.9 kg/m^2^), overweight (23–24.9 kg/m^2^), and obese (≥25 kg/m^2^)^27^. Of Korean nationals, 96% are of Korean origin and most Korean immigrants have an Asian background^28^. Therefore, in this study, baseline BMI was ascertained at the time of enrollment and patients were classified into the “overweight/obese group (≥23 kg/m^2^)” or the “normal weight group (18.5–22.9 kg/m^2^)” according to the Asian-Pacific cutoff points.

### Statistical analysis

Categorical variables were presented as numbers and percentages and compared using the chi-square test or Fisher’s exact test. For continuous variables, the normal distribution of each dataset was confirmed using the Kolmogorov–Smirnov test. Continuous variables with normal distribution were presented as the mean ± standard deviation and compared using the independent sample t-test. Continuous variables without normal distribution were presented as median (interquartile range) and compared using the Mann-Whitney test. The incidence of the endpoints was displayed with Kaplan-Meier curve and differences were assessed using the log-rank test. In addition, multivariate Cox proportional-hazards regression analysis was used to identify independent predictors of the primary endpoint. Factors entered into the multivariate analysis included those with p values < 0.10 in the univariate analysis and variables with known prognostic value. To adjust for the uneven distribution of baseline characteristics, propensity-score analyses were performed. Variables selected for the propensity score were: age, sex, hypertension, diabetes mellitus, dyslipidemia, history of CAD, history of thyroid disease, current smoker, CCBs, beta-blockers and RAS inhibitors. Statistical analyses were performed using SPSS version 20.0 (IBM Corporation, Armonk, NY, USA) and the R programming language, version 2.12 (R Foundation for Statistical Computing), and p values < 0.05 were considered statistically significant.
